# Facial diplegia as the first manifestation of Burkitt lymphoma with a Guillain-Barré syndrome–like presentation: a case report

**DOI:** 10.1186/s12883-026-05153-4

**Published:** 2026-07-18

**Authors:** Irem Yagmur Demir, Ahmet Kasim Kilic, Banu Ozen Barut

**Affiliations:** Department of Neurology, Kartal Dr. Lutfi Kirdar City Hospital, University of Health Sciences, Istanbul, Türkiye

**Keywords:** Facial diplegia, Burkitt lymphoma, Guillain-Barré syndrome, GBS-like syndrome, Central nervous system involvement, Case report

## Abstract

**Background:**

Guillain-Barré syndrome (GBS) is among the most common causes of acute inflammatory polyneuropathy and may present with cranial nerve involvement, including facial diplegia. Rarely, hematologic malignancies can produce a GBS-compatible or GBS-like neurological phenotype, creating a diagnostic challenge in the acute setting.

**Case presentation:**

A 50-year-old immunocompetent woman presented with bilateral facial paralysis, dysarthria, dysphagia, areflexia, and mild left upper-limb weakness. Cerebrospinal fluid (CSF) analysis showed albuminocytologic dissociation. Baseline electromyography and nerve conduction studies (EMG/NCS), performed early in the course, showed non-specific sensorimotor polyneuropathy without definitive demyelinating features. Because the clinical syndrome and CSF findings were compatible with a time-sensitive working diagnosis of GBS, intravenous immunoglobulin was initiated. Follow-up EMG/NCS in the second week evolved to show demyelinating features with secondary axonal involvement, compatible with GBS. However, rapidly progressive leukocytosis, markedly elevated lactate dehydrogenase and ferritin levels, profound weight loss, splenomegaly, para-aortic lymphadenopathy, and blast-like cells on peripheral smear prompted parallel hematologic reassessment. Peripheral blood flow cytometry was consistent with Burkitt lymphoma, and repeat CSF flow cytometry demonstrated a CD10 + germinal center-derived B-cell neoplastic population, indicating central nervous system involvement. The patient subsequently deteriorated with aspiration pneumonia, sepsis/shock, and a fatal course before lymphoma-directed chemotherapy could be initiated.

**Conclusions:**

Burkitt lymphoma with central nervous system involvement can present with a GBS-compatible acute neuropathic phenotype, including facial diplegia. The initial diagnosis of GBS may be clinically reasonable in a time-sensitive setting; however, major systemic and hematologic red flags should prompt early parallel evaluation for malignancy. Peripheral blood and CSF flow cytometry may provide decisive diagnostic evidence when biopsy or further work-up is not feasible.

## Introduction

Guillain–Barré syndrome (GBS) is a common cause of acute neuropathy and may present with cranial nerve involvement, including facial diplegia [1, 2]. However, a broad differential diagnosis should be considered, especially when the clinical course is unusual or laboratory findings are atypical, since diagnostic anchoring may delay recognition of alternative or additional etiologies [3–5]. Hematologic malignancies can produce GBS-like phenotypes through several mechanisms, including direct nerve infiltration (neurolymphomatosis), leptomeningeal dissemination, or, less commonly, paraneoplastic immune-mediated neuropathy [6, 7]. Neurolymphomatosis and malignant leptomeningeal involvement may be difficult to identify clinically and radiologically, and CSF flow cytometry can provide important diagnostic evidence in appropriate contexts [8–11].

We present a case of Burkitt lymphoma with bilateral facial diplegia, bulbar symptoms, areflexia, and CSF findings initially compatible with GBS, in which the subsequent course revealed major systemic and hematologic red flags. The educational value of this case is not that the initial GBS diagnosis was unreasonable, but rather that a GBS-compatible presentation may require parallel reassessment for malignancy when the systemic context becomes discordant with uncomplicated GBS.

## Case presentation

A 50-year-old woman presented with left facial pain and numbness, left postauricular pain, dysarthria, and dysphagia. Her medical history was notable for psychosis diagnosed 5 years earlier and type 2 diabetes mellitus treated with insulin and oral antidiabetic agents. Approximately 10–12 days before admission, she developed postrenal acute kidney injury due to bilateral grade 2–3 hydronephrosis and underwent bilateral double-J ureteral stent placement (Table 1). Neurological symptoms began on the seventh day after the procedure. She also reported pelvic pain and vaginal bleeding; gynecologic evaluation revealed uterine fibroids, and total abdominal hysterectomy with bilateral salpingo-oophorectomy was recommended. She had unintentional weight loss of approximately 20 kg over 1.5–2 months. Her medications included insulin, aripiprazole, olanzapine, and valproic acid.

**Table 1:** Chronological timeline of clinical course, investigations, and management

Time point	Key events / symptoms	Key findings / investigations	Management / decisions
Weeks–months before admission	Progressive systemic symptoms	~ 20 kg unintentional weight loss over 1.5–2 months	—
Day 1 urology admission	Postrenal acute kidney injury due to grade 2–3 hydronephrosis	Bilateral hydronephrosis	Bilateral double-J ureteral stent placement
Day 7 (neurology referral)	Acute/subacute facial diplegia, dysarthria, and dysphagia	Bilateral facial weakness, bulbar signs, lower-limb areflexia, and mild left upper-limb weakness	Neurological evaluation
Day 7	Central nervous system imaging	Cranial CT and contrast-enhanced brain MRI: no acute or structural lesion	—
Day 7	Baseline electrophysiology	Early non-specific sensorimotor polyneuropathy; sural SNAP abnormalities and reduced CMAPs; no definitive demyelinating pattern	Interpreted in the clinical context of suspected GBS
Day 7	Lumbar puncture	CSF: 0 cells; protein 80.6 mg/dL (reference range, 0–45 mg/dL); glucose 68 mg/dL; albuminocytologic dissociation	IVIG started for time-sensitive suspected GBS
Day 7	Infection work-up context after recent urology admission and stenting	Marked leukocytosis and elevated CRP/procalcitonin	Empiric meropenem initiated while cultures were pending
Days 7–11	Treatment period	—	IVIG 2 g/kg total over 5 days
Day ~ 12	Infectious reassessment	Blood cultures negative; urine culture grew Candida spp.; stool tests negative	Meropenem discontinued because there was no evidence of bacterial infection
Day 10–12	Delayed CSF results	OCB type 3; cytology showed lymphocytes and occasional monocytes	OCB interpreted as non-specific and not decisive for the initial treatment decision
Day 13–14	Follow-up electrophysiology	Repeat EMG/NCS: demyelinating features emerged with secondary axonal involvement, compatible with GBS	Electrophysiological evolution supported the GBS-compatible phenotype
Hospital course	Escalating hematologic red flags	WBC increased to 114,000/µL; LDH and ferritin markedly elevated; peripheral smear showed blast-like cells	Urgent hematologic evaluation and flow cytometry
After hematologic work-up	Diagnostic confirmation	Peripheral blood flow cytometry: Burkitt lymphoma; repeat CSF flow cytometry: CD10 + germinal center-derived B-cell neoplasia, indicating CNS involvement	Transfer to hematology for urgent management
Late course	Rapid deterioration	Hypotension, hypoxemia, aspiration pneumonia, and sepsis/shock	ICU care; fatal outcome

*Abbreviations*: *AKI* acute kidney injury, *CMAP* compound muscle action potential, *CRP* C-reactive protein, *CSF* cerebrospinal fluid, *EMG/NCS* electromyography/nerve conduction studies, *GBS* Guillain–Barré syndrome, *ICU* intensive care unit, *IVIG* intravenous immunoglobulin, *LDH* lactate dehydrogenase, *OCB* oligoclonal band, *SNAP* sensory nerve action potential, *WBC* white blood cell count

During a urology follow-up visit, bilateral facial weakness was noted, and she was referred to neurology. On examination, she was alert, oriented, and cooperative. Speech was dysarthric. She had bilateral facial paralysis: she was unable to raise her eyebrows, eyelid closure was incomplete (1–2 mm gap), and Bell’s phenomenon was present. The gag reflex was reduced; palatal elevation was symmetric; and the uvula deviated to the right. Motor examination showed proximal weakness (Medical Research Council [MRC] 3/5) and distal weakness (MRC 4/5) in the left upper extremity, with normal strength (MRC 5/5) in both lower extremities. Deep tendon reflexes were hypoactive in the upper extremities and absent in the lower extremities.

Cranial CT and contrast-enhanced brain MRI revealed no significant abnormalities. EMG/NCS performed on the seventh day of symptom onset showed a decreased right sural sensory nerve action potential (SNAP) and an absent left sural SNAP, along with decreased compound muscle action potentials (CMAPs) in both peroneal nerves and the left tibial nerve. F-wave latencies and conduction velocities were within normal limits. This baseline study was interpreted as an early non-specific sensorimotor polyneuropathy rather than definitive electrodiagnostic GBS. CSF analysis showed no cells, elevated protein (80.6 mg/dL; reference range, 0–45 mg/dL), and glucose 68 mg/dL, consistent with albuminocytologic dissociation. Laboratory testing revealed marked leukocytosis (white blood cell count [WBC] 36,000/µL; neutrophils 19,640/µL; lymphocytes 8,780/µL), lactate dehydrogenase (LDH) 4,443 U/L, ferritin 1,264 ng/mL, C-reactive protein (CRP) 110 mg/L, and procalcitonin 0.9 ng/mL. Serologic testing for HBsAg, anti-HBs, anti-HCV, and anti-HIV was negative. Anti-ganglioside antibody testing was not performed due to lack of consent.

Given the acute onset, bilateral facial diplegia, bulbar symptoms, areflexia, and CSF albuminocytologic dissociation, GBS was considered the initial time-sensitive working diagnosis. The patient was therefore treated with intravenous immunoglobulin (IVIG) at a total dose of 2 g/kg over 5 days.

Because of elevated inflammatory markers and the recent urological procedure with ureteral stenting, an infectious diseases consultation recommended empiric meropenem (1 g three times daily), and blood and urine cultures were obtained. After five days of antibiotic therapy, laboratory tests showed further progression of hematologic abnormalities (WBC 48,000/µL and LDH 6,007 U/L), while CRP decreased to 64 mg/L. Blood cultures were negative; urine culture grew Candida spp.; and stool testing for parasites and Clostridioides difficile toxin was negative. Meropenem was discontinued. The leukocyte count continued to rise, and peripheral smear revealed approximately 5% blast-like cells and increased granulocytic precursors.

Follow-up EMG/NCS performed in the second week showed decreased bilateral peroneal CMAPs, slowed conduction velocities, decreased tibial conduction velocity with an absent tibial F-wave, and decreased right median F-wave persistence. Needle EMG of the facial nerves showed rare denervation. These findings were compatible with an acute/subacute demyelinating polyneuropathy with secondary axonal involvement, fulfilling electrophysiological criteria compatible with GBS [12, 13].

CSF oligoclonal banding later demonstrated a type 3 pattern; this result became available approximately 10–12 days after lumbar puncture. CSF cytology showed lymphocytes and occasional monocytes.

A contrast-enhanced abdominopelvic CT performed earlier during the evaluation of hydronephrosis and pelvic symptoms had revealed splenomegaly and para-aortic lymphadenopathy. CA-125 and CA 15 − 3 levels were elevated. A paraneoplastic panel and flow cytometry were ordered; the paraneoplastic panel was negative. The WBC count subsequently increased to 114,000/µL. Peripheral blood flow cytometry performed at an external laboratory was reported as consistent with Burkitt lymphoma. Repeat lumbar puncture with CSF flow cytometry demonstrated a CD10 + germinal center-derived B-cell neoplastic population, indicating central nervous system involvement. The patient was transferred to the hematology service. She later developed hypotension and desaturation, required intensive care unit admission, and progressed to aspiration pneumonia and sepsis/shock. Despite supportive care, a fatal course occurred before lymphoma-directed chemotherapy could be initiated.

## Discussion

### Initial diagnostic reasoning and electrophysiological evolution

Bilateral facial diplegia or other cranial neuropathies can occur in GBS [1, 2]. In this case, the initial decision to treat for GBS was clinically reasonable, given the combination of acute/subacute bilateral facial paralysis, bulbar symptoms, areflexia, and CSF albuminocytologic dissociation. Because GBS is a time-sensitive diagnosis, IVIG was appropriately initiated as a working diagnosis requiring early treatment [14, 15].

The electrophysiological findings are best interpreted temporally. The baseline EMG/NCS was performed early and was non-specific rather than definitively diagnostic. Importantly, sural nerve abnormalities or an early axonal pattern do not exclude GBS, particularly when studies are performed early in the disease course [16]. The follow-up study later evolved to show demyelinating features with secondary axonal involvement, supporting a GBS-compatible presentation [12, 13]. Thus, the case should not be framed as a simple false GBS diagnosis, but as a GBS-overlapping clinical syndrome in which subsequent systemic and hematologic abnormalities required parallel reassessment.

CSF findings also required cautious interpretation. Albuminocytologic dissociation can support GBS, but it is not specific and may also be seen in inflammatory, infectious, paraneoplastic, or malignant disorders involving nerve roots or meninges [17, 18]. Similarly, the type 3 oligoclonal band pattern was a non-specific finding in this context. It became available approximately 10–12 days after lumbar puncture and therefore did not influence the initial decision to initiate IVIG. Given the confirmed CNS involvement, the patient’s remote history of psychosis may raise the possibility of earlier, clinically unrecognized neuropsychiatric involvement; however, contemporaneous neurological assessment from that period was unavailable, and this point remains speculative.

### When to reconsider GBS and investigate for malignancy

The stronger diagnostic signal was the combination of systemic and hematologic red flags: rapidly rising leukocyte count, markedly elevated LDH and ferritin levels, profound weight loss, splenomegaly, para-aortic lymphadenopathy, and blast-like cells on peripheral smear. These abnormalities are difficult to explain by uncomplicated GBS alone and should prompt early hematologic evaluation, including peripheral blood flow cytometry and, when CNS involvement is suspected, CSF flow cytometry.

Lack of improvement after IVIG was an additional warning sign, although it should not be interpreted retrospectively as proof against GBS by itself. More convincing was the convergence of progressive hematologic abnormalities and systemic features disproportionate to a straightforward inflammatory neuropathy or isolated infection. In this patient, peripheral blood and repeat CSF flow cytometry established Burkitt lymphoma with CNS involvement. Despite diagnostic confirmation, the patient deteriorated rapidly with aspiration pneumonia, hypoxemia, and sepsis/shock, which limited further work-up and precluded timely chemotherapy.

### Mechanistic interpretation of lymphoma-related neuropathy

The mechanisms of lymphoma-related neuropathy should be interpreted according to the strength of evidence. The best-supported mechanism in this case is leptomeningeal/CNS involvement, demonstrated by CSF flow cytometry showing a CD10 + germinal center-derived B-cell neoplastic population. Flow cytometry can add diagnostic value in suspected leptomeningeal dissemination and may identify malignant CNS involvement even when routine imaging is unrevealing [11].

Direct peripheral nerve infiltration or neurolymphomatosis is a plausible but unproven contributor, suggested by the cranial neuropathy phenotype and the known ability of lymphoma to involve peripheral nerves [8–10]. However, direct peripheral nerve infiltration was not proven because nerve biopsy and FDG-PET were not performed. A paraneoplastic or immune-mediated neuropathy remains a less-supported alternative mechanism in this case, although the clinical and electrophysiological overlap with GBS means that the immune-mediated possibility cannot be completely excluded.

For this reason, the term “GBS-like” is used to describe a neurological phenotype that substantially overlapped with acute inflammatory polyradiculoneuropathy. It is not intended to imply that GBS was unreasonable at presentation or that the case was simply “not GBS” from the outset.

### Comparison with reported cases

Representative published lymphoma cases with GBS-like features are summarized in Table 2 [19–21]. These reports show that the overlap can arise through more than one pathway. Some patients appear to have true or probable immune-mediated GBS occurring in association with lymphoma, whereas others—particularly those with cranial multineuritis, root enhancement, malignant cells in CSF, or pathological evidence of nerve infiltration—are better explained by direct lymphomatous involvement of the peripheral nervous system or leptomeninges.

**Table 2:** Representative published lymphoma cases with GBS-like features

Study	Underlying malignancy	GBS-like presentation	Ancillary findings / favored mechanism	Outcome / clinical lesson
Fitzgerald and Stephens, 2020 [19]	Burkitt lymphoma with primary neurolymphomatosis	Acute cranial multineuritis / cranial neuropathies	Findings supported direct neural infiltration and secondary CNS involvement as the key explanation	Illustrates that Burkitt lymphoma may first appear as a cranial neuropathy syndrome rather than classic systemic lymphoma
Colombo et al. 2025 [20]	Primary peripheral nervous system diffuse large B-cell lymphoma / neurolymphomatosis	Acute polyradiculoneuritis of cranial nerves and lumbar roots	Most GBS criteria were initially met, including electrophysiology and albuminocytologic dissociation; final diagnosis was established at autopsy. Favored mechanism: direct lymphoma infiltration	Highlights that even convincing GBS-like presentations may conceal occult lymphoma
Petrakis et al. 2025 [21]	Hodgkin lymphoma	Paresthesias, paraparesis, diplopia, later facial palsy and tetraparesis	CSF albuminocytologic dissociation and mainly motor demyelinating polyradiculoneuritis; possible immune/paraneoplastic mechanism	Neurological symptoms improved after lymphoma-directed therapy; suggests that some lymphoma-associated cases may represent true or probable immune-mediated GBS

GBS-like refers to cases in which the presenting neurological syndrome substantially overlapped with an acute inflammatory polyradiculoneuropathy phenotype; not all reports represent pathologically proven GBS, and some may reflect malignant leptomeningeal or peripheral nerve involvement rather than a purely immune-mediated neuropathy

The present case adds to this spectrum by showing that Burkitt lymphoma with CNS involvement may initially appear clinically compatible with GBS, while the evolving hematologic profile reveals the need for urgent malignant reassessment.

## Limitations

This report has several limitations. Anti-ganglioside antibody testing was not performed because consent was not obtained. Although such testing might have provided additional supportive information for a GBS-spectrum diagnosis, it would not necessarily have changed the overall interpretation of the case, given the subsequent confirmation of Burkitt lymphoma with CNS involvement. Direct peripheral nerve infiltration was not pathologically proven because nerve biopsy and FDG-PET were not performed. Finally, the patient’s rapid deterioration prevented additional diagnostic procedures and lymphoma-directed chemotherapy.

## Conclusion

This case illustrates that Burkitt lymphoma with CNS involvement can present with facial diplegia and a GBS-compatible acute neuropathic phenotype in an immunocompetent adult. The initial decision to treat for GBS may be clinically reasonable when the presentation is time-sensitive and supported by CSF and evolving electrophysiological findings. However, rapidly progressive leukocytosis, markedly elevated LDH and ferritin levels, profound weight loss, lymphadenopathy, splenomegaly, blast-like cells on peripheral smear, or deterioration despite appropriate immunotherapy should prompt urgent parallel evaluation for hematologic malignancy. Peripheral blood and CSF flow cytometry can be decisive in establishing the diagnosis when biopsy or further imaging is not feasible.

## Data Availability

All data generated or analyzed during this study are included in this published article. Additional de-identified clinical details are available from the corresponding author on reasonable request, subject to institutional and privacy restrictions.

## Abbreviations

AKI: Acute kidney injury
CMAP: Compound muscle action potential
CRP: C-reactive protein
CSF: Cerebrospinal fluid
EMG/NCS: Electromyography/nerve conduction studies
FDG-PET: Fluorodeoxyglucose positron emission tomography
GBS: Guillain-Barré syndrome
ICU: Intensive care unit
IVIG: Intravenous immunoglobulin
LDH: Lactate dehydrogenase
MRC: Medical Research Council
OCB: Oligoclonal band
SNAP: Sensory nerve action potential
WBC: White blood cell count
